# Cholinergic transmission is impaired in patients with idiopathic normal-pressure hydrocephalus: a TMS study

**DOI:** 10.1007/s00702-019-02036-6

**Published:** 2019-06-21

**Authors:** Raffaele Nardone, Stefan Golaszewski, Kerstin Schwenker, Francesco Brigo, Miriam Maccarrone, Viviana Versace, Luca Sebastianelli, Leopold Saltuari, Yvonne Höller

**Affiliations:** 1 Department of Neurology, Franz Tappeiner Hospital, Via Rossini 5, 39012 Merano, BZ Italy; 2 Department of Neurology, Christian Doppler Klinik, Paracelsus Medical University, Salzburg, Austria; 3 Karl Landsteiner Institut für Neurorehabilitation und Raumfahrtneurologie, Salzburg, Austria; 4 Department of Neuroscience, Biomedicine and Movement Science, University of Verona, Verona, Italy; 5 Department of Neurorehabilitation, Hospital of Vipiteno, Vipiteno, Italy; 6 Research Department for Neurorehabilitation South Tyrol, Bolzano, Italy; 7 Department of Neurology, Hochzirl Hospital, Zirl, Austria; 8 Department of Psychology, University of Akureyri, Akureyri, Iceland

**Keywords:** Idiopathic normal-pressure hydrocephalus, Transcranial magnetic stimulation, Cholinergic transmission, Short latency afferent inhibition

## Abstract

The pathophysiological mechanisms of cognitive and gait disturbances in subjects with normal-pressure hydrocephalus (NPH) are still unclear. Cholinergic and other neurotransmitter abnormalities have been reported in animal models of NPH. The objective of this study was to evaluate the short latency afferent inhibition (SAI), a transcranial magnetic stimulation protocol which gives the possibility to test an inhibitory cholinergic circuit in the human brain, in subjects with idiopathic NPH (iNPH). We applied SAI technique in twenty iNPH patients before ventricular shunt surgery. Besides SAI, also the resting motor threshold and the short intracortical inhibition to paired stimulation were assessed. A significant reduction of the SAI (*p* = 0.016), associated with a less pronounced decrease of the resting motor threshold and the short latency intracortical inhibition to paired stimulation, were observed in patients with iNPH at baseline evaluation. We also found significant (*p* < 0.001) correlations between SAI values and the gait function tests, as well as between SAI and the neuropsychological tests. These findings suggest that the impairment of cholinergic neurons markedly contributes to cognitive decline and gait impairment in subjects with iNPH.

## Introduction

Idiopathic normal-pressure hydrocephalus (iNPH) is characterized by the classic Adams triad of cognitive dysfunction, urinary incontinence, and gait impairment. Gait and balance disorders are the leading presentations, whereas cognitive decline and incontinence appear as the disease progresses (Williams and Relkin 2013). Improvement in walking performance following a large-volume lumbar puncture (CSF tap test) is the key selection criterion for ventricular shunt surgery and for predicting responsiveness to the shunt therapy. Despite the clinical importance of the symptoms, the pathophysiological mechanisms of iNPH cognitive and gait disturbances remain unclear and objective methods for its assessment are lacking.

In fact, the degree of ventriculomegaly does not predict the level of behavioral impairment in NPH (Del Bigio et al. 2003) nor does the regression of ventriculomegaly after shunting necessarily correspond to the degree of clinical improvement (Del Bigio et al. 1997a, b).

Disturbed cholinergic neurotransmission, together with delayed hippocampal neuronal death and accumulation of beta-amyloid may contribute to memory dysfunction (Kondziella et al. 2008).

On the other hand, cholinergic dysfunction was found to be an important contributor to gait dysfunction in subjects with Parkinson’s disease (PD) (Rochester et al. 2012), a condition that is similarly characterized by loss of balance, slowness and small steps, although NPH patients perform worse (Bugalho et al. 2013).

A transcranial magnetic stimulation (TMS) protocol, the short latency afferent inhibition (SAI), may give direct information about the function of some cholinergic pathways in the human motor cortex.

SAI is significantly reduced in Alzheimer’s disease (AD) (Di Lazzaro et al. 2002, 2004, 2005; Nardone et al. 2008) and can be increased by acetylcholinesterase inhibitors (Di Lazzaro et al. 2005).

We hypothesize that SAI assessment might allow the contribution of cholinergic dysfunction to cognitive decline and gait disturbances to be evaluated. Therefore, we aimed to evaluate in the present study SAI in a group of subjects with NPH, and its correlation with cognitive and gait impairments.

## Materials and methods

### Patients

Twenty patients with a clinical and neuroimaging diagnosis of iNPH were recruited consecutively for the study. Patients were included in the study if they met criteria for probable iNPH (Relkin et al. 2005) and were capable of cooperating sufficiently for neurophysiological testing. The diagnosis of iNPH was based on the presence of the following symptoms and signs: (1) gradually developing gait disturbance; (2) cognitive deterioration, urinary incontinence, or both; (3) progression of symptoms over time; (4) a CT/MR imaging study showing ventricular enlargement (Evans index > 0.3), with transependymal diffusion and no significant cortical atrophy; (5) normal CSF pressure at lumbar puncture; and (6) improvement of clinical symptoms after lumbar tap test or following continuous external lumbar CSF drainage over 2 days. Exclusion criteria were as follows: (1) any spinal cord lesion that might cause myelopathy or severe lumbar radiculopathy, as verified by neuroimaging and EMG evaluation; (2) history of head trauma with documented brain damage in the last year that might affect excitatory and inhibitory responses to TMS; (3) CNS disease that might explain the clinical symptoms; (4) history or evidence of conditions that might cause secondary hydrocephalus; (5) diabetic polyneuropathy; and (6) pacemaker, metallic implants, or any other contraindication to TMS as specified in the safety guidelines for that procedure (Rossi et al. 2009).

A total of 20 patients (8 women and 12 men; mean age 73.6 years) fulfilled inclusion and exclusion criteria and were enrolled in the study.

Twenty age-matched neurologically healthy subjects (9 women and 11 men; mean age 74.1 years) constituted the control group.

Global cognitive function was examined by means of the Mini-Mental Scale Examination (MMSE, Folstein et al. 1975). Learning and memory were evaluated by means of the Rey Auditory Verbal Learning Test (RAVLT), executive functions by means of the Digit Span and the Trail Making Test (TMT) B, psychomotor speed by means of the TMT A. For reference and description of these tests see Lezak et al. (2004).

In addition, gait abnormalities were quantified by the Timed Up and Go Test (TUG, Podsiadlo and Richardson 1991) and the total walking distance for the 6-min walk test (6MWT, ATS Committee on Proficiency Standards for Clinical Pulmonary Function Laboratories 2002).

Based on CT and MR imaging studies, ventricular enlargement was assessed using an Evans index defined as the maximum width of the frontal horns divided by the maximum inner width of the skull.

The demographic characteristics, as well as the clinical und neurophysiological findings, are shown in the Table 1.

**Table 1:** Demographic characteristics, clinical and neurophysiological data of the patients with idiopathic normal-pressure hydrocephalus and the control subjects

	Patients	Controls
Age (year)	73.6 ± 8.06	74.8 ± 8.03
TUG (s)	18.85 ± 4.81	14.6 ± 2.52
6MWT	240.55 ± 31.68	610 ± 37.73
MMSE	25.7 ± 3.55	29.55 ± 0.6
RAVLT	27.65 ± 9.41	60.04 ± 6.31
Digit span	7.7 ± 1.92	4.1 ± 0.91
TMT-A	268.7 ± 39.77	125.45 ± 27.48
TMT-B	111.7 ± 38.26	49.5 ± 8.38
RMT^a^	49 ± 11.7	51.5 ± 11.8
SICI^b^	37.45 ± 10,01	34.7 ± 11.13
SAI^b^	64.75 ± 18.1	43.15 ± 9.65

Data are expressed as mean values ± SD

*Y* years, *s* seconds, *TUG* timed up and go test, *6MWT* total walking distance for the 6-min walk test, *MMSE* mini-mental scale examination, *RAVLT* rey auditory verbal learning test, *TMT* trail making test, *RMT* the resting motor threshold, *SICI* the short latency intracortical inhibition, *SAI* short latency afferent inhibition

^a^% of maximum stimulator output

^b^% of test response

### Transcranial magnetic stimulation

TMS was performed using a High-power Magstim 200 magnetic stimulator (Magstim Co., Whitland, Dyfed, UK) connected to a Bistim module throughout all measurements. A figure-of-eight coil with external loop diameters of 9 cm was held over the motor cortex at the optimum scalp position to elicit motor responses in the first dorsal interosseous (FDI) muscle. The dominant hemisphere was selected for stimulating patients and healthy subjects. The induced current flows in a postero-anterior direction. Motor evoked potentials (MEPs) were recorded via two 9-mm diameter Ag–AgCl electrodes with the active electrode applied over the motor point of the muscle and the reference on the metacarpophalangeal joint of the index finger. MEPs were amplified and filtered (bandwidth 3–3000 Hz) by D150 amplifiers (Digitimer, Welwyn Garden City, Hertfordshire, UK).

We evaluated the following TMS parameters: the resting motor threshold (RMT), the short latency intracortical inhibition (SICI) to paired TMS, and the SAI.

RMT was defined as the minimum stimulus intensity that produced a minimal motor evoked response (about 50 µV in 50% of ten trials) at rest (Rossini et al. 2015).

SICI was studied using the technique of Kujirai et al. (1993). Two magnetic stimuli were given through the same stimulating coil over the motor cortex and the effect of the first (conditioning) stimulus on the second (test) stimulus was investigated. The intensity of the conditioning stimulus was set to 80% RMT; the second (test) shock intensity was adjusted to evoke a MEP in relaxed FDI with an amplitude of approximately 1 mV, peak-to-peak. The timing of the conditioning shock was altered in relation to the test shock. Inhibitory interstimulus intervals (ISIs) of 2, 3, and 5 ms were investigated. Ten stimuli were delivered at each ISI also for test stimulus and single MEP. For these recordings, muscle relaxation is very important and the subject was given audiovisual feedback at high gain to assist in maintaining complete relaxation. The presentation of conditioned and unconditioned trials was randomized. The amplitude of the conditioned EMG responses was expressed as the percentage of the amplitude of the test EMG responses. The amplitudes of the conditioned responses were averaged obtaining grand mean amplitudes of the three inhibitory ISIs.

SAI was studied using the technique of Tokimura et al. (2000). Conditioning stimuli were single pulses (200 ms) of electrical stimulation (with the cathode positioned proximally), applied through bipolar electrodes to the median nerve at the wrist. The intensity of the conditioning stimuli was set at just over motor threshold for evoking a visible twitch of the thenar muscles. The intensity of the test cortical magnetic shock was adjusted to evoke a muscle response in relaxed FDI with an amplitude of approximately 1 mV peak-to-peak. The conditioning stimulus to the peripheral nerve preceded the test magnetic cortical stimulus. ISIs were determined relative to the latency of the N20 component of the somatosensory evoked potential evoked by stimulation of the median nerve. In right-handed subjects, the active electrode for recording the N20 potential was attached 3 cm posterior to C3 (10–20 system), and the reference was 3 cm posterior to C4 (vice versa for left-handed subjects). Five hundred responses were averaged to identify the latency of N20 peak. ISIs from the latency of the N20 component plus 2 ms to the latency of the N20 component plus 8 ms were investigated in steps of 1 ms. Eight stimuli were delivered at each ISI also for test stimulus and single MEP. We calculated the average of the MEP obtained after cortical magnetic stimulation alone, and the average of the MEP obtained by conditioning cortical magnetic stimulus with a peripheral stimulus to the median nerve at the wrist at the seven different ISIs studied. The amplitude of the conditioned MEP was expressed as a percentage of the amplitude of the test MEP. The percentage inhibition of the conditioned responses at the seven different ISIs was averaged to obtain a grand mean. Subjects were given audio–visual feedback at high gain to assist in maintaining complete relaxation.

### Statistical analysis

Statistics were carried out using the software environment R (R Core Team 2013), including the package npmv which is available at http://CRAN.R-project.org/package=npmv and is based on the nonparametric multivariate inference methodology described by Bathke et al. (2008).

We included group (patients vs. controls) as a between-subject factor and as within-factors (repeated measures; response variables) age, RMT, SAI, SICI, TUG, 6MWT, MMSE, RAVLT, Digit Span, TMT-A, and TMT-B. Together with the test, we provide also relative effects. The relative effects can be understood as tendencies expressed as estimated probabilities.

We also evaluated the correlation between SAI and the gait function tests (TUG and 6MWT), as well as between SAI and the neuropsychological tests (MMSE, RAVLT, TMT A and B, Digit Span) for the two groups. Spearman correlation was used for this purpose. Because of 14 separate correlations, we used Bonferroni correction to interpret the results.

To control whether SAI correlates with age, we performed again a Spearman correlation for the whole group.

## Results

We found a significant main effect of group (*F*(5.28,200.59) = 28.13; *p* < 0.001). The closed-loop post hoc test shows that the main effect of group is significant for SAI, 6MWT, MMSE, RAVLT, Digit Span, TMT A, and TMT B, but not for age, RMT, SICI, and TUG.

**Fig. 1:**
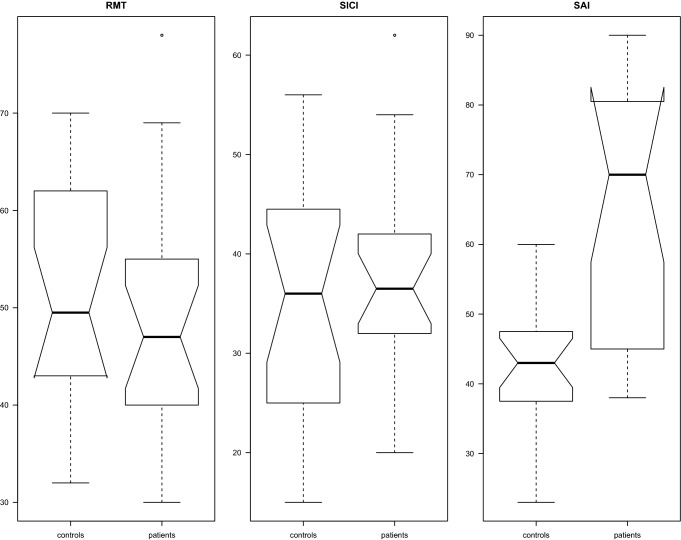
The distributions of RMT, SICI, and SAI are shown for the two groups are shown. As the boxplots reveal, the distributions overlap largely for RMT and SICI, resulting in no significant difference, while there is minimal overlap for SAI, resulting in a significant difference between groups. *MSO* maximum stimulator output

Figure 1 shows the distributions of RMT, SICI, and SAI for the two groups.

Relative effects indicate that the probability that a randomly chosen participant from the patient group exhibits a larger SAI than a randomly chosen participant from the control group is high with 0.81, and for 6MWT it is null (Table 2).

**Table 2:** Relative effects for factor group

	Controls	Patients
Age	0.52	0.48
SAI	0.19	0.81
RMT	0.58	0.42
SICI	0.46	0.54
TUG	0.24	0.76
6MWT	1.00	0.00
MMSE	0.94	0.06
RAVLT	0.99	0.01
DigitSpan	0.06	0.94
TMT A	0.01	0.99
TMT B	0.00	1.00

*SAI* short latency afferent inhibition, *RMT* the resting motor threshold, *SICI* the short latency intracortical inhibition, *TUG* timed up and go test, *6MWT* total walking distance for the 6‐min walk test, *MMSE* mini-mental scale examination, *RAVLT* rey auditory verbal learning test, *TMT* trail making test

**Fig. 2:**
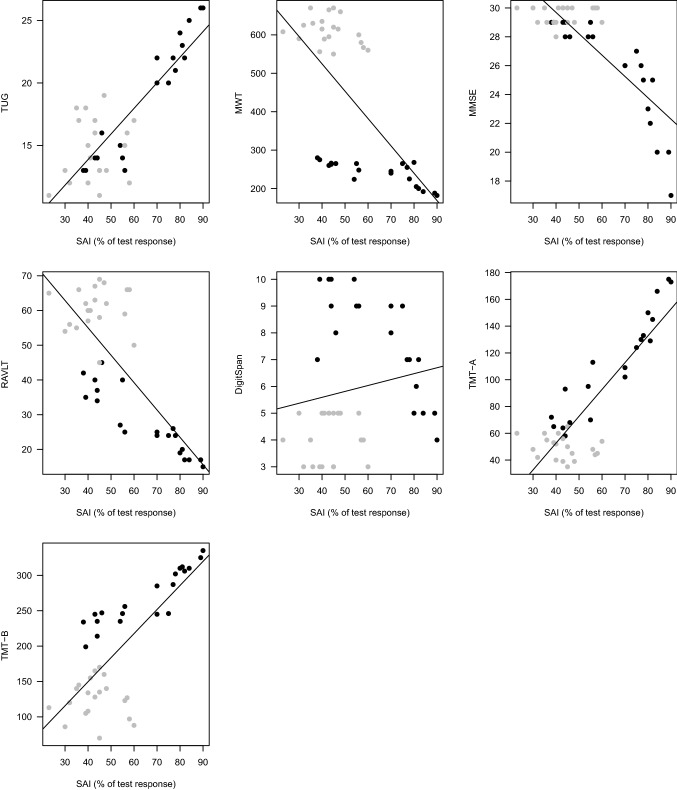
Scatterplots of SAI vs. TUG, 6MWT, MMSE, RAVLT, digit span, TMT-A, and TMT-B. The line represents the regression line, fitted to the data. SAI correlates significantly with all gait and neuropsychological tests. *MMSE* mini-mental scale examination, *RAVLT* rey auditory verbal learning test, *TMT* trail making test, *TUG* timed up and go test, *6MWT* total walking distance for the 6-min walk test

The correlations are shown in Table 3 and illustrated in Fig. 2. All variables (TUG, 6MWT, RAVLT, Digit Span, TMT A and TMT B) correlated with SAI in patients, but not in control subjects. After Bonferroni correction for multiple conduction of the correlation test, these correlations were still significant (*p* divided by 14 correlations: *p* < 0.003).

**Table 3:** Correlations separately for the two groups

	Controls		Patients	
	Rho	*p* value	Rho	*p* value
TUG	0.11	0.64	0.92	< 0.001
6MWT	− 0.27	0.24	− 0.76	< 0.001
MMSE	0.12	0.61	− 0.95	< 0.001
RAVLT	0.19	0.43	− 0.92	< 0.001
DigitSpan	0.12	0.62	− 0.79	< 0.001
TMT A	− 0.29	0.22	0.94	< 0.001
TMTB	0.02	0.93	0.93	< 0.001

*TUG* timed up and go test, *6MWT* total walking distance for the 6-min walk test, *MMSE* mini-mental scale examination, *RAVLT* rey auditory verbal learning test, *TMT* trail making test

SAI did not correlate significantly with age (rho = 0.25; *p *= 0.12).

## Discussion

The main and novel finding of this study was a significant reduction of SAI, a surrogate measure of cholinergic activity. Also SICI and RMT were decreased, similar to that reported in a previous TMS study (Chistyakov et al. 2012), even if in our study the differences did not reach statistical significance. Methodological issues could account for this discrepancy. In particular, in the previous study single- and paired-pulse TMS have been performed over leg motor area.

The present findings are consistent with previous experimental studies. So far, the only valid animal model of NPH is the chronic adult kaolin hydrocephalus (Khan et al. 2006). Adult rats with chronic kaolin hydrocephalus show cognitive impairment such as decreased learning and spatial memory, as well as other symptoms including gait ataxia and bradykinesia comparable to NPH patients (Del Bigio et al. 1997a, b, 2002, 2003).

In the adult kaolin hydrocephalus complex neurotransmitter disturbances, including the cholinergic system (Tashiro et al. 1997a; Egawa et al. 2002), have been described.

Progressive injury to cholinergic systems (Tashiro et al. 1997a, b; Egawa et al. 2002), in combination with the delayed neuronal death in hippocampus (Klinge et al. 2003), may contribute to hydrocephalic dementia. In particular, progressive hydrocephalus results in functional injuries of cholinergic and GABAergic neurons in the neostriatum and dopaminergic neurons in the substantia nigra compacta by mechanical distortion (Tashiro et al. 1997a).

In another experimental study in Wistar neonatal rats, the number of cholinergic neostriatal neurons was significantly reduced at 2 and 4 weeks in the acute form, at 8 weeks in the subacute form of hydrocephalus (Ishizaki et al. 2000).

The impairment of spatial memory in kaolin-induced hydrocephalic rats is associated with dysfunction of the hippocampal cholinergic and noradrenergic systems (Egawa et al. 2002).

The disturbance in balance of these neurotransmitter systems in the basal ganglia may explain some motor functional disabilities in hydrocephalus.

On the other hand, pathological, neuroimaging, and clinical evidence suggest that degeneration of cholinergic systems may contribute to impaired balance and gait in PD (Mancini et al. 2015).

Interestingly, significant associations were found between SAI, gait speed, inhibition, age and postural instability and gait disorder score in subjects with PD (but not control subjects) (Rochester et al. 2012). Regression analysis showed that reduced SAI was an independent predictor of slower gait speed in participants with PD.

It should be noted that SAI did not correlate in our study with age, therefore, the abnormal SAI values in our iNPH patients cannot be related only to the deterioration of the cholinergic system seen in normal human cognitive aging (Young-Bernier et al. 2014; Di Lorenzo et al. 2016).

The major limitation of our preliminary study was that we cannot exclude a concomitant AD pathology, which is present (neuritic plaques) in over 40% of patients (Golomb et al. 2000). Notably, there is a relationship between amyloid pathology (elevated amyloid precursor protein-derived proteins) and iNPH (Jeppsson et al. 2013) as well as between SAI and CSF beta-amyloid levels in AD (Martorana et al. 2012). It has been hypothesized, that AD and NPH might represent the extremes in a cluster of disorders characterized by a continuum of CSF circulatory failure with subsequent neurodegeneration (Silverberg et al. 2003). Indeed, many NPH cases remain with severe cognitive and motor deficits after shunting, even when ventricular size decreases postoperatively (Vanneste 2000; Kondziella et al. 2008).

Interestingly, in a recent paper SAI has been correlated to gait disturbances also in AD patients (Schirinzi et al. 2018), shedding light on the role of cholinergic system in cognitive motor control (Pelosin et al. 2016). Several studies are thus highlighted the usefulness of SAI as a marker of gait in cognitive impaired people. Conversely, SAI has been correlated to cognitive functions (especially memory more than executive function) in a sample of cognitively unimpaired old and young subjects (Young-Bernier et al. 2012; Bonnì et al. 2017) but not in AD patients (Koch et al. 2015). It should also be considered that patients with iNPH show a specific pattern of impairment on tests sensitive to frontostriatal dysfunction, which is distinct from that exhibited by patients with mild AD (Iddon et al. 1999).

In conclusion, the present study provides neurophysiological evidence that disturbances in balance of cholinergic neurotransmitter system may partly explain dementia and motor functional disabilities in subjects with NPH. It would be of great interest to examine in future studies the impact of shunt surgery on these neurophysiological findings.
